# Trajectories of functional limitations, health-related quality of life and societal costs in individuals with long COVID: a population-based longitudinal cohort study

**DOI:** 10.1136/bmjopen-2024-088538

**Published:** 2024-11-13

**Authors:** Jiunn Wang, Henry Goodfellow, Sarah Walker, Ann Blandford, Paul Pfeffer, John R Hurst, David Sunkersing, Katherine Bradbury, Chris Robson, William Henley, Manuel Gomes

**Affiliations:** 1 Department of Primary Care and Population Health, University College London, London, UK; 2 Royal Free Hospital, London, UK; 3 University of Exeter Medical School, Exeter, UK; 4 Department of Computer Science, University College London, London, UK; 5 Barts and Royal Free London NHS Trust, London, UK; 6 Respiratory Medicine, University College London, London, UK; 7 Institute of Health Informatics, University College London, London, UK; 8 Institute for Life Sciences, University of Southampton, Southampton, UK; 9 Living With Health, London, UK; 10 Health Statistics Group, University of Exeter, Exeter, UK

**Keywords:** COVID-19, Post-Acute COVID-19 Syndrome, Fatigue, Quality of Life, Health Care Costs, Observational Study

## Abstract

**Abstract:**

**Objectives:**

To examine trajectories of functional limitations, fatigue, health-related quality of life (HRQL) and societal costs of patients referred to long COVID clinics.

**Design:**

A population-based longitudinal cohort study using real-time user data.

**Setting:**

35 specialised long COVID clinics in the UK.

**Participants:**

4087 adults diagnosed with long COVID in primary or secondary care deemed suitable for rehabilitation and registered in the Living With Covid Recovery (LWCR) programme between 4 August 2020 and 5 August 2022.

**Main outcome measures:**

Generalised linear mixed models were fitted to estimate trajectories of functional limitations, using the Work and Social Adjustment Scale (WSAS); scores of ≥20 indicate moderately severe limitations. Other outcomes included fatigue using the Functional Assessment of Chronic Illness Therapy–Fatigue (FACIT-F) reversed score (scores of ≥22 indicate impairment), HRQL using the EQ-5D-5L, and long COVID-related societal costs, encompassing healthcare costs and productivity losses.

**Results:**

The mean WSAS score at 6 months after registration in the LWCR was 19.1 (95% CI 18.6, 19.6), with 46% of the participants (95% CI 40.3%, 52.4%) reporting a WSAS score above 20 (moderately severe or worse impairment). The mean change in the WSAS score over the 6-month period was −0.86 (95% CI −1.32, –0.41). The mean reversed FACIT-F score at 6 months was 29.1 (95% CI 22.7, 35.5) compared with 32.0 (95% CI 31.7, 32.3) at baseline. The mean EQ-5D-5L score remained relatively constant between baseline (0.63, 95% CI 0.62, 0.64) and 6 months (0.64, 95% CI 0.59, 0.69). The monthly societal cost per patient related to long COVID at 6 months was £931, mostly driven by the costs associated with working days lost.

**Conclusions:**

Individuals referred to long COVID clinics in the UK reported small improvements in functional limitations, fatigue, HRQL and ability to work within 6 months of registering in the LWCR programme.

**STRENGTHS AND LIMITATIONS OF THIS STUDY**

- Prospective, longitudinal follow-up of a large population of individuals with a confirmed long COVID diagnosis and real-time outcome data collection.
- Sample includes individuals referred to 35 long COVID clinics, geographically spread across the UK, enhancing the generalisability of the results.
- The use of generic, validated measures facilitates the interpretation of the long COVID burden compared with other diseases.
- All analyses were based on complete cases; we acknowledge that individual dropouts may have introduced bias.
- Separate trajectory models for individuals with different follow-up periods were conducted to minimise biases due to dropout.

## Introduction

 Long COVID, as defined by the National Institute for Health and Care Excellence, refers to the persistence of symptoms lasting for at least 12 weeks following COVID-19 infection.^1^ It has been suggested that at least 1 in 10 patients who had COVID-19 experiences long COVID symptoms.^2^ By March 2024 (latest figures made available by the Office for National Statistics), an estimated 2 million people in the UK (3.3% of the population) reported experiencing symptoms consistent with long COVID.^3^ These symptoms have a particularly significant impact on the working-age population, leading to both absenteeism (productivity loss due to time off work) and presenteeism (lower productivity due to illness while working).^4^ It has been estimated that these productivity losses amounted to £5.7 billion in the UK between 2022 and 2023.^5^

The manifestations of long COVID are diverse and vary significantly among individuals. Common symptoms include fatigue, cognitive impairment, breathlessness, anxiety and depression.^6^ These symptoms impair individuals’ daily functioning, including their ability to work, manage home responsibilities, engage in social and leisure activities, and maintain personal relationships. The high prevalence of long COVID, the diversity of the symptoms and rehabilitation pathways have added to the complexity of providing adequate care for long COVID patients in the UK National Health Service (NHS).^3^

To help address these challenges, a bespoke digital health intervention, Living With Covid Recovery (LWCR), was developed and implemented across 35 long COVID clinics in the UK.^7^ The LWCR intervention was designed to facilitate remote rehabilitation and to support the recovery of people living with long COVID. The LWCR collected patient-reported outcome measures (PROMs), encompassing the aspects of breathlessness, fatigue, anxiety, cognition and depression, enabling clinicians to monitor and adjust the care provided to each patient.^8^

In a recent cross-sectional study,^7^ we described the characteristics and self-reported symptoms of a cohort of patients referred to long COVID clinics in England. We found that over half of this care-seeking population reported moderately severe or worse functional limitations within the first month (baseline) of registering in the LWCR programme. Fatigue seemed to be the dominant symptom explaining the variation in functional limitations, with a substantial impact on the individual’s ability to work and activities of daily living.

A recent umbrella review suggested that long COVID is likely to have an impact on family life, social functioning and mental health but highlighted that the existing evidence is premature and insufficient to inform healthcare decision-making.^9^ They emphasised that representative, prospective studies are vital to improve the current understanding of the impact of long COVID on functional impairment, health outcomes and costs over time.

This paper reports on the trajectories of functional limitations, fatigue, health-related quality of life (HRQL) and societal costs within the first 6 months after registration in the LWCR. In addition, we investigate whether these trajectories differ according to patient characteristics or the extent of the individuals’ participation in the LWCR programme.

## Methods

### Design and setting

This is a population-based, longitudinal cohort study of patients with long COVID, who were referred to 35 specialised long COVID clinics in the UK and participated in the LWCR programme.

### Intervention

The LWCR was a digital health intervention designed to support the recovery of individuals living with long COVID symptoms. The LWCR programme was developed collaboratively by a multidisciplinary team including clinicians, patient and public involvement (PPI) representatives, academics and an industry partner, and encompasses three key components: (1) a mobile app for patients, which collects their symptoms and uses that information to deliver tailored, personalised advice; (2) a dashboard that allows clinicians to review patient’s progress and communicate with them and (3) a clinical pathway that specifies how patients can safely receive this remote supported care. Patient information collected through the LWCR app enables the long COVID clinics to manage the high volume of patients and provide remote supported care. Further details about the development of the LWCR intervention are reported elsewhere.^7 8^

### Population

In 2020, the UK NHS established specialist clinics to provide multidisciplinary services for individuals with a diagnosis of long COVID referred from primary and secondary care. Eligible participants were identified by the long COVID clinic as being suitable for the LWCR programme if they were aged 18 or over, had access to a smartphone, were considered likely to benefit from the digital intervention, were working, fit for rehabilitation and able to read English. This study included individuals with long COVID registered in the LWCR programme between 4 August 2020 and 5 August 2022, who completed the baseline questionnaires for the outcomes of interest within 1 month of registration (defined as ‘baseline’ in this study). Individuals could complete the questionnaires independently or supported by a health professional in the long COVID clinic. We also have those individuals who are either retired or have chosen not to work for reasons unrelated to long COVID. The paper reported the results for those individuals who have not ticked this box.

### Primary outcome

The primary outcome was the trajectory of Work and Social Adjustment Scale (WSAS) between baseline and 6 months after registration in the LWCR. WSAS is a self-reported measurement of functional limitations, evaluating how a specific condition affects a patient’s ability to carry out activities across five dimensions: (1) work, (2) home management, (3) social leisure activities, (4) private leisure activities and (5) close relationships.^10^ Each domain is rated between 0 (not at all) and 8 (very severely), resulting in a total score ranging from 0 to 40, with higher scores indicating greater functional impairment. A WSAS score of 20 or more has been considered to indicate moderately severe or worse functional impairment.^10 11^

### Secondary outcomes

Secondary outcomes for this study included trajectories of fatigue, HRQL and long COVID-related societal costs.

#### Functional Assessment of Chronic Illness Therapy–Fatigue

The Functional Assessment of Chronic Illness Therapy–Fatigue (FACIT-F) measures self-reported fatigue and its impact on daily activities and function. This measure consists of 13 items, with each item rated between 0 (very much fatigue) and 4 (not at all). The summary score ranges from 0 to 52, with lower scores indicating more severe levels of fatigue. A threshold value of 30 was chosen to indicate impairment, in line with fatigue reported in a cancer population.^12^ In this study, we reversed the FACIT-F score (calculated as 52 minus the reported score) to align the direction of the score with that of the primary outcome. We refer to this as Reversed FACIT-F Scale; higher values of the reversed scale represent greater fatigue, with scores equal or above 22 indicating impairment.

#### EQ-5D-5L

The EuroQol Five Dimensions (EQ-5D-5L) is a self-reported HRQL measure consisting of five domains: mobility, self-care, usual activities, pain/discomfort and anxiety/depression. Each domain is rated on a 5-level scale. Take mobility, for example, patients can choose from five options, ranging from ‘no problems in walking about’ to ‘unable to walk about’. Responses to these questions are then combined with health-related preference weights from the UK population to construct an index EQ-5D-5L score.^13^ This score is anchored at 0 (death) and 1 (full health), allowing for negative values indicating health states worse than death.

#### Costs

The cost analysis took the societal perspective and included healthcare costs across primary and secondary care settings, as well as costs associated with working days lost (productivity losses) due to long COVID. The health service use questionnaire in the LWCR app recorded the number of general practitioner visits, psychotherapy and physiotherapy sessions, hospital inpatient stays, and outpatient appointments in the past 4 weeks (month). To calculate healthcare costs, we combined the resource use with unit costs from the Unit costs of Health and Social Care^14^ and NHS national tariffs.^15^ The health service use questionnaire also asked patients to report the number of days off work due to long COVID in the past 4 weeks. To calculate the total cost associated with working days lost, we costed the number of days off work using the national average hourly pay (£13.57) and the average working hours per week (33.9 hours) estimated by the Office for National Statistics.^16^

#### Patient demographics

The LWCR also included a Patient Demographic Questionnaire, which collected sociodemographic data from patients, including age, gender, ethnicity and highest educational level. The Index of Multiple Deprivation (IMD) is derived from the individual’s postcode.

### Patient and public involvement

This study had a substantial PPI with coinvestigator (KB), the steering group, individual work package management groups and an overall PPI Advisory Group. The feedback from PPI was essential at an early stage in determining the PROMs chosen in the study and the primary outcome measure of the WSAS and at a later stage in interpreting the findings of the study and implications for practice.

### Statistical analysis

The WSAS, fatigue and EQ-5D-5L scores were summarised as means for each month following registration in the LWCR over the 6-month follow-up. Trajectories were reported by fitting linear mixed models of the monthly means on the month following registration in the LWCR, assuming a quadratic relationship between the outcome and time. Sociodemographic variables, including age, gender, ethnicity, highest educational level and IMD, were also included in the models as control variables. The mean change from baseline to 6 months in the WSAS score was modelled using an analysis of covariance.

We reported monthly mean costs up to 6 months following registration, based on the resource use reported on a monthly basis. Linear mixed-effects models were also used to estimate the cost trajectories, adjusting for the same sociodemographic and time variables included in the outcome trajectory models. In addition, we also investigated whether the probability of reporting any working days lost at 6 months was associated with individual characteristics and time off work at baseline.

All our analyses were based on complete cases assuming that the dropout was at random conditional on the casemix. The frequency with which each questionnaire (one for each endpoint) was completed differed according to endpoint, and hence the sets of complete cases varied across endpoints. We reported differences in sociodemographic factors between individuals with different follow-up times. To address potential biases arising from these differences, we reported separate trajectories for three types of participants: (1) those who completed the questionnaires only within the first month of registration (baseline), (2) those who completed the questionnaire at baseline and follow-up between 2 and 5 months after registration in the LWCR and (3) those who completed the questionnaire at baseline and follow-up until at least 6 months after registration in the LWCR.

## Results

### Patient demographics

The study included 4087 individuals with long COVID, who completed the baseline Patient Demographic Questionnaire within the first month of registration in the LWCR (table 1). There were missing data on education (n=272, 6.7%), ethnicity (n=990, 24%) and IMD (n=316, 7.7%). The participants had a mean age of 47.3 (12.2) years, 3859 (94%) of whom were in the working-age bracket of 18–65 years. The participants were 71% (n=2920) female, 89% (n=3365) of white ethnicity, and about half (n=2001, 52%) had a degree or postgraduate degree. 10% (n=385) were from the most deprived quintile and 24% (n=919) from the least deprived. Similar patient characteristics were seen in those who completed the baseline WSAS, EQ-5D-5L and resource use questionnaires (online supplemental table S1).

**Table 1:** Baseline sociodemographic characteristics of the study participants for the whole sample, and according to completion status of the primary outcome

Patient characteristicsn (%), unless stated otherwise	Study populationn (%)* (n=4087)	WSAS completed within the first month of registration (baseline) only n (%)*(n=2228)	WSAS completed at baseline and follow-up between 2 and 5 months after registration n (%)*(n=949)	WSAS completed at baseline and follow-up until at least 6 months after registration n (%)* (n=341)
Age (years), mean (SD)	47.3 (12.2)	46.3 (12.3)	48.5 (11.9)	49.7 (12.0)
Age category (years)				
18–30	429 (10.5)	262 (11.8)	83 (8.7)	26 (7.6)
30–50	1960 (48.0)	1118 (50.2)	431 (45.4)	142 (41.6)
50–65	1470 (36.0)	738 (33.1)	383 (40.4)	146 (42.8)
65 and over	228 (5.6)	110 (4.9)	52 (5.5)	27 (7.9)
Gender				
Female	2920 (71.4)	1584 (71.1)	692 (72.9)	239 (70.1)
Male	1155 (28.3)	638 (28.6)	253 (26.7)	101 (29.6)
Non-binary	12 (0.2)	5 (0.2)	4 (0.4)	1 (0.3)
Highest educational level				
No education	151 (4.0)	90 (4.2)	35 (3.7)	14 (4.1)
School leaver (NVQ 1–2)	847 (22.2)	479 (22.1)	213 (22.5)	85 (25.1)
A-level (NVQ-3)	816 (21.4)	480 (22.1)	179 (18.9)	82 (24.2)
Degree (NVQ-4)	771 (20.2)	414 (19.1)	206 (21.8)	62 (18.3)
Postgraduate degree (NVQ-5)	1230 (32.2)	701 (32.4)	313 (33.1)	96 (28.3)
Missing	272	64	3	2
Ethnicity				
White	3365 (88.8)	1898 (88.4)	846 (89.7)	313 (92.9)
Non-white	426 (11.2)	249 (11.6)	97 (10.3)	24 (7.1)
Missing	990	81	6	4
IMD quintile				
1–2 (20% most deprived)	385 (10.2)	210 (9.8)	114 (12.2)	33 (9.9)
3–4	748 (19.8)	425 (19.9)	181 (19.4)	67 (20.1)
5–6	875 (23.2)	488 (22.9)	206 (22.0)	90 (27.0)
7–8	844 (22.4)	489 (22.9)	199 (21.3)	75 (22.5)
9–10 (20% least deprived)	919 (24.4)	523 (24.5)	235 (25.1)	68 (20.4)
Missing	316	93	14	8

* Percentages are based on the complete cases within each variable.

IMD, Index of Multiple DeprivationNVQ, National Vocational Qualification; WSAS, Work and Social Adjustment Scale

Table 1 compares the characteristics of the participants in the whole sample (n=4087) with those with different completion status of the WSAS questionnaire. Among individuals who completed the baseline WSAS (n=3518) within the first month of registration, 63% (n=2228) completed the questionnaire only at baseline, 27% (n=949) completed it at baseline and follow-up between 2 and 5 months after registration, and 10% (n=341) completed it at baseline and follow-up until at least 6 months after registration.

Participants who completed WSAS questionnaire at baseline and follow-up between 2 and 5 months after registration were on average older (48.5 vs 46.3), more likely to have a degree (54.9% vs 51.5%) and more likely to be female (72.9% vs 71.1%) compared with those who completed the WSAS questionnaire at baseline only. Participants with follow-up until at least 6 months (vs those who completed the WSAS questionnaire at baseline only) were on average older (49.7 vs 46.3), less likely to have a degree (46.6% vs 51.5%), more likely to be white (92.9% vs 88.4%) and less likely to be in the least deprived quintile (20.4% vs 24.5%).

### Functional limitations

**Figure 1:**
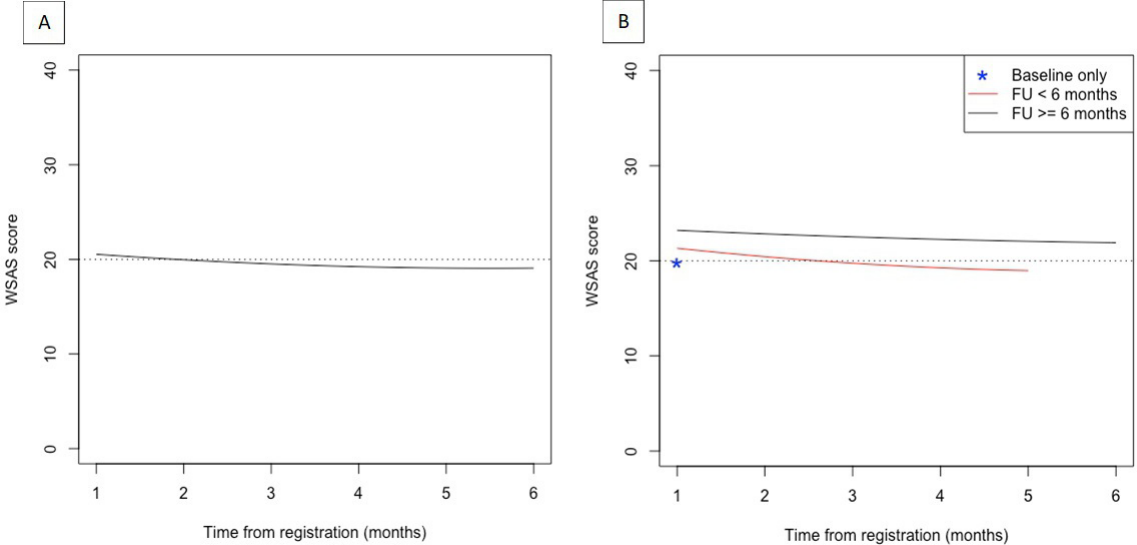
(**A**) Estimated trajectories of the Work and Social Adjustment Scale (WSAS) over the 6-month follow-up (FU). (**B**) Observed trajectories according to completion status of the WSAS questionnaire: (1) WSAS completed within the first month of registration (baseline) only; (2) WSAS completed at baseline and FU between 2 and 5 months after registration and (3) WSAS completed at baseline and FU until at least 6 months after registration. A WSAS score of 20 (dashed line) or more is considered to indicate moderately severe or worse functional impairment.

Figure 1 reports the trajectories of the functional limitations for all respondents and according to the completion status of the WSAS questionnaire. The estimated mean WSAS score at 6 months after registration based on the complete case analysis (N=3518; figure 1A) was 19.1 (95% CI 18.6, 19.6), with nearly half of the respondents (46.3%, 95% CI 40.3%, 52.4%) expected to have a WSAS score above 20 (moderately severe or worse impairment). This compares to a mean WSAS score of 20.5 (95% CI 20.2, 20.9) at baseline, at which point 54.9% (95% CI 51.7%, 58.0%) of the participants had a WSAS score above 20. The mean change in the WSAS score over the 6-month period was −0.86 (95% CI −1.32, –0.41). Similar trajectories were observed for each individual component of the WSAS (online supplemental figure S2). In particular, functional impairment in the individual’s ability to work and enjoy social leisure activities remains moderately severe or worse over the 6- month follow-up.

The mean WSAS score over time was somewhat higher (greater impairment) for those participants who completed WSAS at baseline and follow-up until at least 6 months after registration, compared with those with shorter follow-up (figure 1B). The observed mean (SD) baseline WSAS score for the participants who reported WSAS at baseline only was 19.8 (SD 10.1; n=2228). This compared with a mean baseline WSAS score of 21.3 (SD=9.1; n=949) and 23.3 (SD=9.1; n=341) for those who reported WSAS at follow-up between 2 and 5 months and at least 6 months after registration, respectively. The slope of the trajectories over time was relatively similar between individuals with follow-up less than 6 months and those with at least 6 months follow-up. Of the participants who completed WSAS at baseline and follow-up until at least 6 months (n=341), 203 (59.5%) reported functional limitations in the moderately severe or worse category (WSAS scores above 20).

Table 2 reports associations between WSAS trajectories over the 6-month follow-up and baseline sociodemographic characteristics. Individuals aged 50 and above reported lower functional impairment over time compared with younger adults. For example, patients aged 65 or above were associated with lower (−4.48, 95% CI −6.18, –2.78) average WSAS score compared with individuals aged 18−29. Men were also associated with a lower average WSAS score (−1.63, 95% CI −2.32, –0.93) over time compared with women. Individuals in the most deprived quintile were associated with a higher (1.41, 95% CI 0.40, 2.43) functional impairment compared with those in quintile 2 or above.

**Table 2:** Associations between the Work and Social Adjustment Scale score trajectories over the 6-month follow-up and baseline sociodemographic characteristics

Patient characteristic	N (%)	Estimate (SE)	95% CI	P value
Age (years)				
18–29	426 (10.5)	Reference		
30–49	1945 (47.9)	−0.73 (0.56)	(−1.82, 0.37)	0.192
50–64	1461 (36.0)	−2.38 (0.57)	(−3.50, 1.25)	<0.001
65+	227 (5.6)	−4.48 (0.87)	(−6.18, 2.78)	<0.001
Gender				
Female	2900 (71.7)	Reference		
Male	1147 (28.3)	−1.63 (0.35)	(−2.32, 0.93)	<0.001
IMD				
Quintiles 2+	3369 (89.8)	Reference		
Quintile 1 (most deprived)	383 (10.2)	1.41 (0.52)	(0.40, 2.43)	0.006
Months since baseline		−0.34 (0.17)	(−0.67, 0.01)	0.041
Age×months since baseline				
18–29		Reference		
30–49		0.14 (0.18)	(−0.21, 0.50)	0.429
50–64		−0.13 (0.18)	(−0.49, 0.22)	0.461
65+		−0.32 (0.25)	(−0.80, 0.17)	0.200

IMD, Index of Multiple Deprivation

### Fatigue

The trajectories of fatigue for all respondents and according to the completion status of the FACIT-F questionnaire are reported in online supplemental figure S1. Similar to the primary outcome, mean reversed FACIT-F scores slightly decreased (less impairment) over time; the estimated mean reversed FACIT-F score at 6 months was 29.1 (95% CI 22.7, 35.5) compared with 32.0 (95% CI 31.7, 32.3) at baseline. However, there were little differences in fatigue trajectories between individuals with different completion status of the FACIT-F questionnaire.

### Health-related quality of life

**Figure 2:**
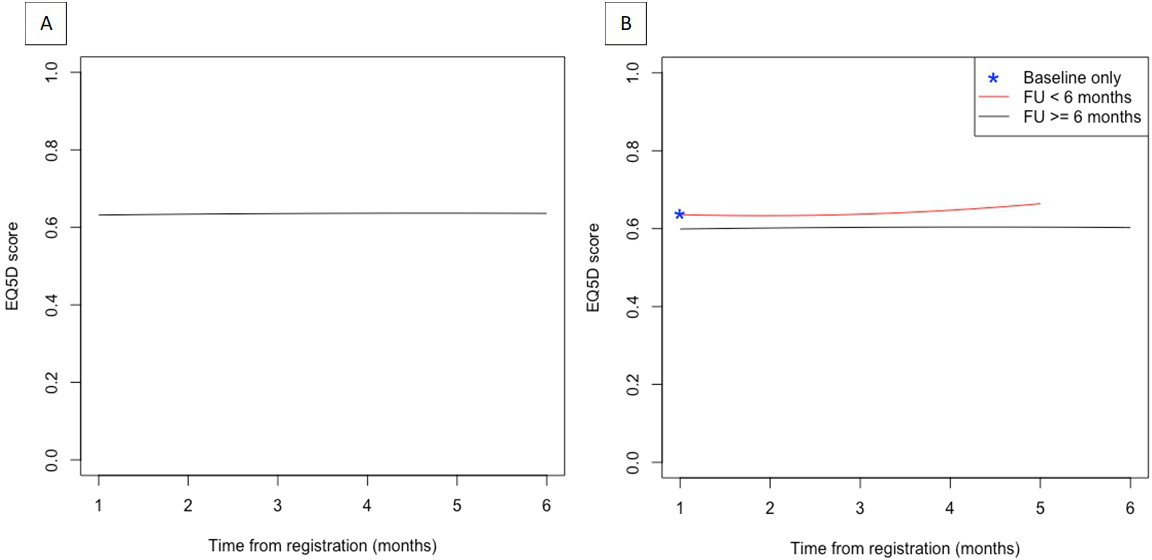
(**A**) Estimated trajectories of the EQ-5D-5L over the 6-month follow-up (FU). (**B**) Observed trajectories according to completion status of the EQ-5D-5L questionnaire: (1) EQ-5D-5L completed within the first month of registration (baseline) only; (2) EQ-5D-5L completed at baseline and FU between 2 and 5 months after registration and (3) EQ-5D-5L completed at baseline and FU until at least 6 months after registration. EQ-5D-5L, EuroQol Five Dimensions.

Figure 2 reports the trajectories of the EQ-5D-5L for all respondents and according to the completion status of the EQ-5D-5L questionnaire. The estimated mean EQ-5D-5L based on the complete case analysis (n=3523) was 0.63 (95% CI 0.62, 0.64) at baseline and 0.64 (95% CI 0.59, 0.69) at 6 months after registration (figure 2A). The differences in the trajectories of EQ-5D-5L between individuals with follow-up less than 6 months and those with follow-up until at least 6 months were small (figure 2B).

### Costs

Of the individuals who completed the baseline resource use questionnaire (n=3422) within the first month of registration, 53% (n=1816) completed the questionnaire only at baseline, 39.4% (n=1347) completed it at baseline and follow-up between 2 and 5 months after registration and 7.6% (n=259) completed it at baseline and follow-up until at least 6 months after registration (online supplemental table S2). Participants with follow-up until at least 6 months (vs those who completed the resource use questionnaire at baseline only) were on average older (50.5 vs 45.8), less likely to have a degree (44.0% vs 52.7%), more likely to be white (93.4% vs 87.6%) and less likely to be in the least deprived quintile (19.5% vs 24.9%).

Table 3 reports resource use and costs according to the completion status of the resource use questionnaire. Full results based on the complete case analysis over the 6-month period are reported in online supplemental table S3. The healthcare costs associated with hospital and primary care visits were small and remained relatively constant over time (online supplemental table S3) and did not differ according to the completion status of the resource use questionnaire (table 3).

**Table 3:** Mean monthly resource use and cost according to completion status of the resource use questionnaire: (1) resource use completed within the first month of registration (baseline) only; (2) resource use completed at baseline and follow-up between 2 and 5 months after registration and (3) resource use completed at baseline and follow-up until at least 6 months after registration

	Resource use completed within the first month of registration (baseline) only (n=1816)		Resource use completed at baseline and follow-up between 2 and 5 months after registration (n=1347)*		Resource use completed at baseline and follow-up until at least 6 months after registration (n=259)	
	Resource useMean (SD)	Cost (£)Mean (SD)	Resource useMean (SD)	Cost (£)Mean (SD)	Resource useMean (SD)	Cost (£)Mean (SD)
GP visits	1.1 (1.4)	42.5 (55.5)	1.0 (1.3)	39.6 (49.5)	0.9 (1.1)	35.9 (42.2)
Outpatient visits	1.0 (1.7)	141.9 (227.3)	0.8 (1.3)	116.0 (182.0)	0.8 (1.4)	113.2 (186.7)
Physiotherapy sessions	0.3 (0.8)	21.2 (53.7)	0.3 (0.7)	20.7 (51.1)	0.4 (1.0)	27.1 (67.1)
Psychotherapy sessions	0.3 (1.0)	9.8 (31.2)	0.3 (0.8)	8.6 (26.6)	0.3 (0.9)	10.9 (29.6)
Inpatient stay (days)	0.0 (0.5)	23.7 (240.1)	0.0 (0.5)	16.8 (240.4)	0.1 (0.5)	31.7 (251.4)
Working day lost	6.2 (9.9)	569.6 (908.2)	6.8 (10.2)	629.3 (937.8)	7.7 (11.1)	712.2 (1016.9)
Total societal cost		808.7 (1053.3)		831.0 (1034.4)		931.1 (1121.8)

* Results reported in this column correspond to a weighted average of the monthly mean resource use and costs across months 2–5.

GP, general practitioner

The participants who completed the questionnaire at baseline only (n=1816) reported on average 6.2 (SD 9.9) days off work due to long COVID within the first month of registration, corresponding to a mean (SD) productivity loss of £570 (908). Almost half of these participants (n=834, 46%) reported at least 1 day off work (per month), and 18% (n=324) reported 20 (or more) days off work at baseline. Individuals who completed the resource at both baseline and any follow-up reported somewhat higher mean working days lost and associated costs. For example, participants who completed the resource use questionnaire at baseline and follow-up until 6 months (n=259) reported 7.7 (11.1) days off work 6 months after registration, which corresponded to a mean (SD) monthly productivity loss at 6 months of £712 (1017). About half of these individuals (n=131, 51%) reported at least 1 day off work, and 23% (n=60) reported 20 or more working days lost at 6 months.

Of those individuals (n=141) who reported at least 1 day off work (per month) at baseline and had follow-up until at least 6 months, 102 (72%) continued to report at least 1 day off work at 6 months, and 51 (36%) reported 20 or more working days lost (unable to work at all). The latter group was particularly worse off at 6 months compared with baseline (online supplemental table S4). Associations between the probability of reporting at least 1 day off work due to long COVID at 6 months and baseline sociodemographic variables are reported in online supplemental material (online supplemental table S5).

## Discussion

### Main findings

This study provides new evidence on the recovery trajectory of care-seeking patients with long COVID and its impact on function, health outcomes and costs. Individuals referred to long COVID clinics in the UK reported little improvements in functional limitations, fatigue and HRQL over the first 6 months after registration in the LWCR programme. Our analysis suggested that almost half of long COVID patients were expected to have moderately severe or worse functional impairment at 6 months, emphasising ongoing challenges in long COVID recovery. Individuals under the age of 50, females and those more deprived appeared to report smaller improvements over time.

Separate analysis according to level of engagement of the individuals in the LWCR programme suggested that individuals with 6 months or longer follow-up reported somewhat higher functional impairment compared with participants with less than 6 months follow-up. However, we found that both groups reported similar, modest improvements over time.

While long COVID-related healthcare utilisation remained relatively constant over the 6-month period, the average number of working days lost increased slightly over time. Almost three-quarters of the participants who reported loss of working days at baseline, and remained engaged in the LWCR programme, continued to report working days lost at 6 months, and over one-third of these were unable to work at all. Societal costs were primarily driven by productivity losses and averaged £931 per individual per month at 6 months after registration.

### Comparisons with previous studies

A previous umbrella review^9^ found that long COVID is likely to have an impact on activities of daily living, social functioning and employment. However, the study concluded that the existing literature provides a patchy, heterogeneous and thus inconclusive picture of the health, social and economic burden of long COVID.

This longitudinal study adds to our recent cross-sectional paper^7^ by showing a persisting functional impairment over the 6-month period after registration in the LWCR. The mean WSAS score was 19.1 (95% CI 18.6, 19.6) at 6 months, suggesting that care-seeking long COVID patients have, on average worse functional impairment than individuals who had a stroke (mean WSAS score of 16).^17^ We found that there was a small improvement in function over time (mean change in WSAS score was −0.86, 95% CI −1.32, –0.41), but this is unlikely to be clinically relevant. A previous study^18^ suggested that a minimal clinically important difference was expected to be 8 points on the 0–40 WSAS scale. In addition, we found that nearly 60% of the participants who remained engaged in the LWCR programme still reported moderately severe or worse impairment at 6 months.

This paper found that the trajectories of the WSAS and FACIT-F scores were remarkably similar over time. This corroborates previous evidence from the cross-sectional study,^7^ which suggested that fatigue was the major driver of the variation in functional limitations. By looking at a much larger sample, this longitudinal analysis finds that HRQL of long COVID patients in the LWCR programme remained persistently low over the 6-month follow-up. The mean EQ-5D-5L at 6 months was 0.64, which is on par with the HRQL observed for patients with advanced cancers.^19 20^

A recent observational study conducted in Switzerland showed that 23% and 17% of patients infected with COVID-19 did not fully recovery by 6 and 24 months, respectively.^21^ This contrasts with our findings, which suggested that about 46% of participants still had moderately severe or worse functional impairment at 6 months. The reasons for this difference are varied and related to differences in the study design and target population. First, the Swiss study included a comparator group (with no COVID infection), which may have helped minimise selection biases. Second, the primary outcome in that study was the EuroQol Visual Analogue Scale (EQ-VAS), which tends to have low sensitivity to small changes in health status and be prone to ceiling effects. Third, the Swiss study included individuals living in a single canton (Zurich) in Switzerland and only those with the wild-type COVID-19 alpha variant, who may be less likely to have prolonged effects over time.^22^

Our findings about more modest levels of improvement over time align well with other European studies reporting on the recovery of long COVID patients.^23^,^25^ A cohort study conducted in the Catalan region followed long COVID patients for a median of 23 months and found that only 8% (26 out of 341) recovered during follow-up.^23^ Another study conducted in Southern Germany also found that about 29% (3289 out of 11 536) of long COVID patients reported impaired general health and working capacity between 6 and 12 months after COVID-19 infection.^24^

Most treatment-seeking long COVID patients in our study sample were under the age of 65 (94%), consistent with existing findings that the prevalence of long COVID was highest among the working-age population.^26 27^ Previous studies indicate between 13% and 43% of COVID-19-affected individuals do not return to work 6 months after infection.^26 28^ Our study shows an average of 7.7 days off work (per month) at 6 months, equivalent to one-third of working days lost. Our study also finds that almost three-quarters of individuals who reported working days lost at baseline continued to report absenteeism at 6 months due to long COVID. This is likely related to symptoms such as fatigue, dyspnoea, cognition and mental health symptoms, as reported elsewhere.^28^,^30^ It is worth noting that we were not able to distinguish between individuals in different types of job arrangements (part time, full time, self-employed), but our PPI work suggested that our sample includes a mix of employment types and hence, it is likely to be representative of the working-age population.

An Australian study suggested that, even for those who returned to work at 6 months, 34% of patients had new problems with mobility, 34% with pain and 43% with usual activities.^31^ This is in line with the findings of a survey conducted by Living With,^32^ which collected feedback from 1874 LWCR participants between March 2023 and March 2024. This survey found that over half (1100 out of 1874) of the individuals who returned to work have struggled at the workplace, and over one-third (650 out of 1874) required adjustment at work. This highlights the prolonged impact of long COVID on the individual’s working ability,^28^ suggesting challenges in job performance even for those returning to work. As a result, our estimated cost associated with working days lost might be an underestimate of the productivity losses due to long COVID as it excludes costs associated with reduced productivity at work (presenteeism).

### Strengths and limitations

One strength of this study lies in the prospective, longitudinal follow-up of a large population of individuals with a confirmed long COVID diagnosis and the real-time data collection on functional impairment, symptoms, HRQL and resource use. The sample includes individuals referred to 35 long COVID clinics in the UK, which have a good geographical representation across the country. In addition, the use of generic, validated measures, such as WSAS and EQ-5D-5L, facilitates the interpretation of the long COVID burden compared with other diseases.

A limitation of our study was the loss to follow-up. We observed that a high proportion of patients disengaged with the LWCR app. As patients had full control over the utilisation of the app, dropout could be attributed to many reasons. A potential concern is that disengagement with the app could be strongly associated with patient’s recovery, in which case, our results would be an overestimate of the overall long COVID burden as they speak to those still needing help. However, the Living With survey^32^ found that only 5% (110 out of 1874) of the respondents reported having stopped using the app because they ‘got better’. In fact, many individuals (nearly 20%) reported that they disengaged with the LWCR app because ‘their symptoms were not improving’. Moreover, this study conducted separate trajectory models for individuals with different follow-up periods and did not find meaningful differences in the trajectories between them.

A related limitation is the potential bias introduced by missing data in the sociodemographic variables. Nevertheless, complete case analysis has been recommended as the primary analysis when low levels of missing data in the explanatory variables were observed compared with dependent variables.^33^

Despite the large and geographically representative sample, individuals recruited to this study were referred by long COVID clinics and needed to meet specific criteria to be eligible to register in the LWCR app. In particular, our sample included individuals who were considered likely to benefit from the LWCR intervention and were fit for rehabilitation. Consequently, our estimates may be somewhat conservative as we potentially excluded individuals less fit and less (digitally) educated than the ‘average’ long COVID patient. In addition, some clinical information, such as the date and severity of the index COVID infection, vaccination status and virus variant, was not available in our study, and hence we were unable to evaluate the extent to which these factors have had an impact on long COVID recovery.

## Conclusion

Individuals referred to long COVID clinics in the UK reported little improvement in functional limitations, fatigue and HRQL over the first 6 months after registration in the LWCR, irrespective of the period that participants remained engaged in the programme. This persistent functional impairment and poor quality of life over time significantly affects the individual’s ability to work and presents a significant economic burden to the individuals themselves as well as to the economy. Addressing ongoing challenges related to fatigue and its impact on work absenteeism should be a priority for future healthcare interventions aimed at supporting the recovery of individuals with long COVID. Important dimensions of health inequality, such as gender and deprivation, should be considered when devising health policy recommendations for the treatment and management of long COVID.

## supplementary material

10.1136/bmjopen-2024-088538 online supplemental file 1

## Data Availability

Data are available on reasonable request.
