# Supplementary material for: Trajectories of functional limitations, health-related quality of life and societal costs in individuals with long COVID: a population-based longitudinal cohort study
Source: BMJ Open. 2024 Nov 13;14(11):e088538. doi: 10.1136/bmjopen-2024-088538 (PMC11574431; doi:10.1136/bmjopen-2024-088538)
Supplement: online supplemental file 1 [file bmjopen-14-11-s001.pdf]

Title: Trajectories of functional limitations, health-related quality of life and societal costs in individuals with Long COVID: a population based longitudinal cohort study

Jiunn Wang[1], Henry Goodfellow[1], Sarah Walker[2], Ann Blandford[3], Paul E Pfeffer[4], John R Hurst[5], David Sunkersing[6], Katherine Bradbury[7], Chris Robson[8], William Henley[2], Manuel Gomes[1].

1. Primary Care and Population Health, University College London, London, UK
2. University of Exeter Medical School, University of Exeter, St Luke's Campus, Exeter, UK.
3. UCL Interaction Centre, University College London, London, UK
4. Department of Respiratory Medicine, Barts Health NHS Trust, London, UK.
5. UCL Respiratory, University College London, London, UK
6. Institute of Health Informatics, University College London, London, UK
7. Psychology, University of Southampton, Southampton, UK
8. Living With, London, UK

SUPPLEMENTARY MATERIALS

Table S1: Baseline socio-demographic characteristics of the study participants for the whole sample, and according to completion status of the baseline questionnaire.

**Patient characteristics n(%), unless stated otherwise**	**Study population n(%)* (n = 4087)**	**WSAS completed n(%)* (n=3518)**	**EQ-5D-5L completed n(%)* (n=3523)**	**Health service use completed n(%)* (n = 3422)**
Age (years), mean (SD)	47.3 (12.2)	47.2 (12.2)	47.3 (12.2)	47.2 (12.1)
Age category (years)				
18 – 30	429 (10.5)	371 (10.5)	369 (10.5)	308 (9.0)
30 - 50	1960 (48.0)	1691 (48.1)	1696 (48.1)	1581 (46.2)
50 - 65	1470 (36.0)	1267 (36.0)	1270 (36.0)	1354 (39.6)
65 and over	228 (5.6)	189 (5.4)	188 (5.3)	179 (5.2)
Gender				
Female	2920 (71.4)	2515 (71.5)	2519 (71.5)	2432 (71.1)
Male	1155 (28.3)	992 (28.2)	993 (28.2)	980 (28.6)
Non-binary	12 (0.2)	10 (0.3)	10 (0.3)	10 (0.3)
Highest educational level				
No education	151 (4.0)	139 (4.0)	132 (3.9)	133 (3.8)
School leaver (NVQ 1-2)	847 (22.2)	777 (22.5)	774 (22.6)	784 (22.3)
A-level (NVQ-3)	816 (21.4)	741 (21.5)	735 (21.5)	766 (21.8)
Degree (NVQ-4)	771 (20.2)	682 (19.8)	685 (20.0)	712 (20.2)
Postgraduate degree (NVQ-5)	1230 (32.2)	1110 (32.2)	1100 (32.1)	1126 (32.0)
Missing	272	69	97	41
Ethnicity				
White	3365 (88.8)	3057 (89.2)	3039 (89.3)	3005 (89.4)
Non-white	426 (11.2)	370 (10.8)	366 (10.7)	365 (10.6)
Missing	990	91	118	61
IMD quintile				
1 to 2 (20% most deprived)	385 (10.2)	357 (10.5)	350 (10.3)	352 (10.5)
3 to 4	748 (19.8)	673 (19.8)	669 (19.8)	671 (20.1)
5 to 6	875 (23.2)	784 (23.0)	777 (23.0)	761 (22.8)
7 to 8	844 (22.4)	763 (22.4)	766 (22.6)	750 (22.4)
9 to 10 (20% least deprived)	919 (24.4)	826 (24.3)	822 (24.3)	808 (24.2)
Missing	316	115	139	80

* Percentages do not include those with missing values in the denominator. WSAS: Work and social adjustment scale. SD: standard deviation. NVQ: National Vocational Qualification. IMD: Index of multiple deprivation.

Figure S1: (A) Estimated trajectories of the Functional Assessment of Chronic Illness Therapy – Fatigue (FACIT-F) reversed score over the 6-month follow up (FU). (B) Observed trajectories

according to completion status of the FACIT-F questionnaire: 1) FACIT-F completed within the first month of registration (baseline) only; 2) FACIT-F completed at baseline and follow-up between two and five months after registration 3) FACIT-F completed at baseline and follow-up until at least six months after registration. Higher values of the reversed FACIT-F score represent greater fatigue, with scores equal or above 22 (dashed line) indicating impairment.

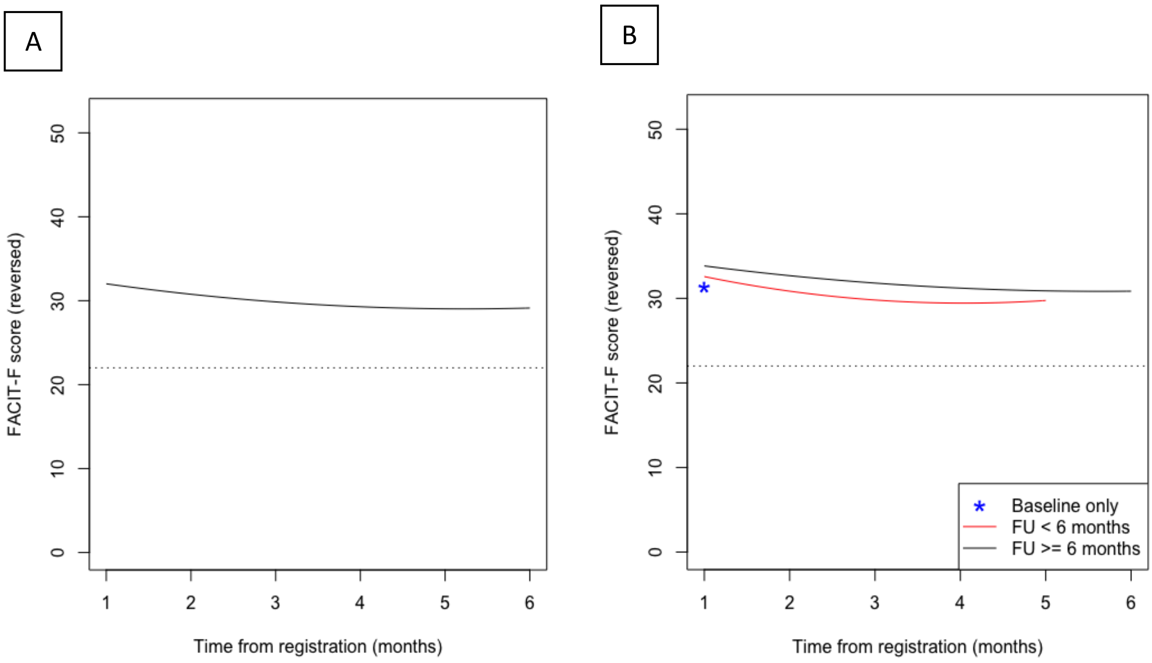

Figure S2: Estimated trajectories of the Work and Social Adjustment Scale (WSAS) individual components over the 6-month follow up. A WSAS score of 4 (dashed line) or more is considered to indicate moderately severe or worse functional impairment.

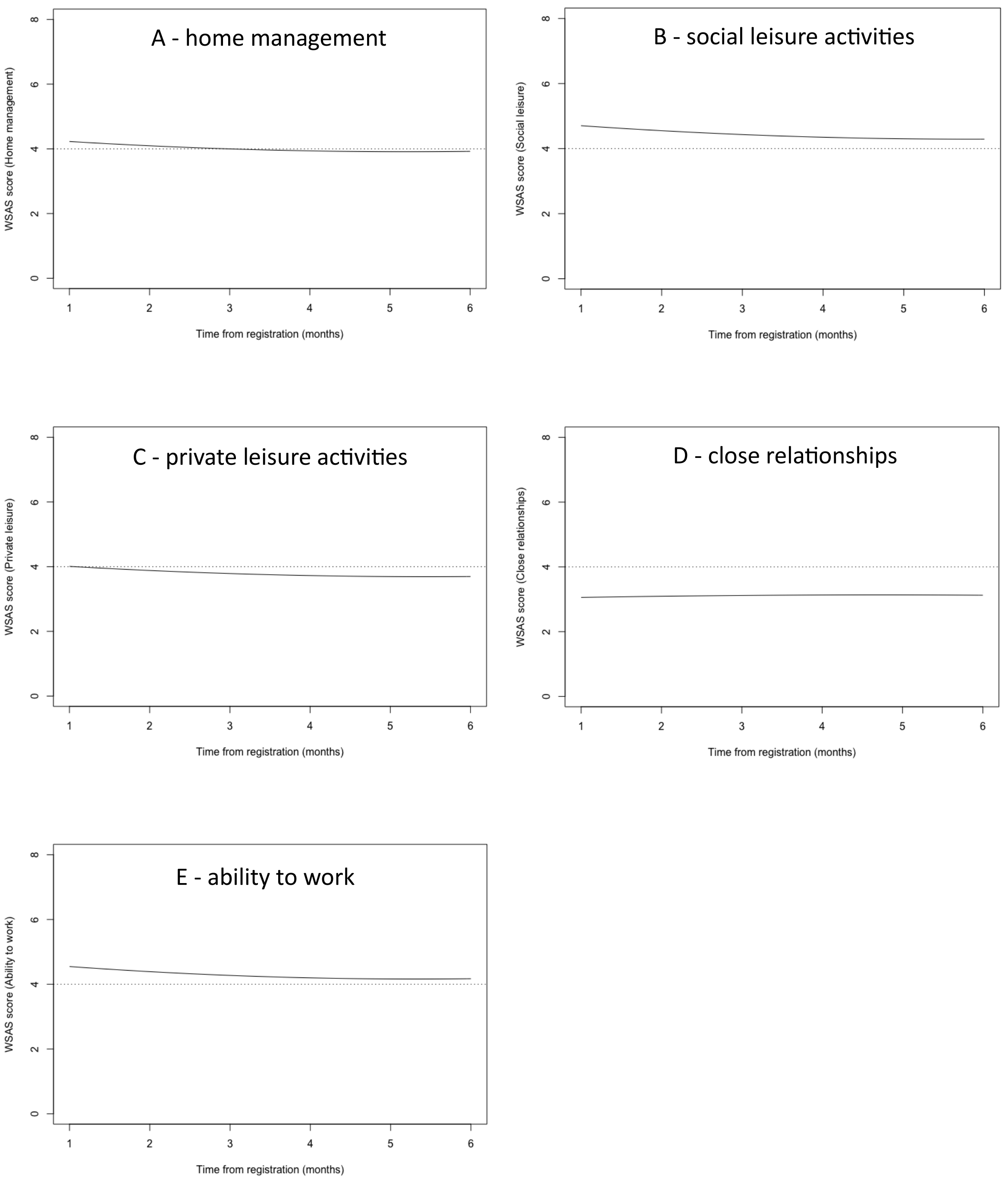

Table S2: Sociodemographic baseline characteristics of the patients in the study, and proportion completed at baseline by completion status of the health service use questionnaire.

**Patient characteristics n(%), unless stated otherwise**	**Resource use completed within the first month of registration (baseline) only n(%)* (n = 1816)**	**Resource use completed at baseline and follow-up between 2 and 5 months after registration n(%)* (n = 1347)**	**Resource use completed at baseline and follow-up until at least 6 months after registration n(%)* (n = 259)**
Age (years), mean (SD)	45.8 (12.4)	48.5 (11.5)	50.5 (11.5)
Age category (years)			
18 - 29	206 (11.3)	87 (6.5)	15 (5.8)
30 - 49	879 (48.4)	611 (45.4)	91 (35.1)
50 - 64	649 (35.7)	570 (42.3)	135 (52.1)
65 and over	82 (4.5)	79 (5.9)	18 (7.0)
Gender			
Female	1266 (69.7)	976 (72.5)	190 (73.4)
Male	546 (30.1)	366 (27.2)	68 (26.3)
Non-binary	4 (0.2)	5 (0.4)	1 (0.4)
Highest educational level			
No education	72 (4.1)	45 (3.3)	11 (4.3)
School leaver (NVQ 1-2)	385 (21.7)	304 (22.6)	70 (27.0)
A-level (NVQ-3)	383 (21.6)	284 (21.1)	64 (24.7)
Degree (NVQ-4)	354 (19.9)	281 (20.9)	49 (18.9)
Postgraduate degree (NVQ-5)	582 (32.8)	432 (32.1)	65 (25.1)
Missing	40	1	0
Ethnicity			
White	1543 (87.6)	1221 (91.0)	241 (93.4)
Non-white	218 (12.4)	121 (9.0)	17 (6.6)
Missing	55	5	1
IMD quintile			
1 to 2 (20% most deprived)	177 (10.1)	150 (11.3)	25 (9.8)
3 to 4	363 (20.7)	251 (18.9)	57 (22.3)
5 to 6	382 (21.7)	313 (23.6)	66 (25.8)
7 to 8	397 (22.6)	295 (22.2)	58 (22.7)
9 to 10 (20% least deprived)	438 (24.9)	320 (24.1)	50 (19.5)
Missing	59	18	3

* Percentages do not include those with missing values in the denominator. SD: standard deviation. NVQ: National Vocational Qualification. IMD: Index of multiple deprivation.

Table S3: Mean monthly resource use and cost for all respondents (complete cases) over the 6-month follow up

	Month 1 (n = 3422)		Month 2 (n = 710)		Month 3 (n = 530)	
	Health service use Mean (SD)	Cost (£) Mean (SD)	Health service use Mean (SD)	Cost (£) Mean (SD)	Health service use Mean (SD)	Cost (£) Mean (SD)
GP visits	1.0 (1.3)	40.6 (52.2)	0.9 (1.1)	34.3 (43.2)	0.9 (1.2)	36.7 (49.0)
Outpatient visits	1.0 (1.5)	131.2 (211.1)	0.7 (1.1)	94.7 (157.0)	0.8 (1.2)	102.9 (162.6)
Physiotherapy sessions	0.3 (0.8)	21.2 (53.6)	0.3 (0.7)	20.1 (48.1)	0.3 (0.6)	19.5 (43.2)
Psychotherapy sessions	0.3 (0.9)	9.4 (30.0)	0.3 (0.8)	10.2 (26.8)	0.3 (0.8)	11.0 (27.6)
Inpatient stay (days)	0.0 (0.4)	19.3 (208.2)	0.0 (0.5)	16.3 (245.5)	0.0 (0.5)	23.7 (241.9)
Working day lost	6.6 (10.1)	609.3 (931.5)	7.0 (10.5)	642.6 (970.1)	7.4 (10.7)	683.9 (985.8)
Total societal cost		831.0 (1050.4)		818.2 (1050.4)		877.4 (1088.0)
	Month 4 (n = 373)		Month 5 (n = 276)		Month 6 (n = 259)	
	Health service use Mean (SD)	Cost (£) Mean (SD)	Health service use Mean (SD)	Cost (£) Mean (SD)	Health service use Mean (SD)	Cost (£) Mean (SD)
GP visits	0.8 (1.0)	32.8 (40.1)	0.9 (1.1)	33.4 (41.8)	0.9 (1.1)	35.9 (42.2)
Outpatient visits	0.7 (1.2)	102.5 (165.7)	0.8 (1.3)	105.7 (175.0)	0.8 (1.4)	113.2 (186.7)
Physiotherapy sessions	0.3 (0.6)	18.7 (44.6)	0.3 (0.7)	18.5 (45.2)	0.4 (1.0)	27.2 (67.1)
Psychotherapy sessions	0.4 (0.9)	11.9 (30.0)	0.4 (0.9)	12.1 (29.2)	0.3 (0.9)	10.9 (29.6)
Inpatient stay (days)	0.0 (0.4)	14.2 (188.5)	0.0 (0.3)	17.5 (158.6)	0.1 (0.5)	31.7 (251.4)
Working days lost	7.4 (10.8)	680.5 (989.1)	7.5 (11.0)	689.7 (1012.3)	7.7 (11.1)	712.2 (1016.9)
Total societal cost		860.7 (1070.3)		876.9 (1086.5)		931.1 (1121.8)

SD: standard deviation. GP: General Practitioner

Table S4. Mean (SD) monthly resource use and costs for the subgroup of individuals (N=51) who reported at least one day off work (per month) at baseline, had follow up until at least 6 months, and reported 20 or more working days lost (unable to work at all).

	Baseline		6-months	
	Resource use Mean (SD)	Cost (£) Mean (SD)	Resource use Mean (SD)	Cost (£) Mean (SD)
GP visits	1.5 (1.4)	60.0 (56.8)	1.3 (1.2)	52.3 (46.9)
Outpatient visits	1.3 (1.3)	174.6 (179.8)	1.5 (2.0)	212.2 (278.5)
Physiotherapy sessions	0.2 (0.4)	10.8 (25.3)	0.4 (0.9)	28.4 (63.5)
Psychotherapy sessions	0.3 (1.2)	10.2 (39.8)	0.2 (0.5)	5.7 (15.5)
Inpatient stay (days)	0.0 (0.0)	0.0 (0.0)	0.1 (0.3)	28.4 (150.0)
Working day lost	22.3 (8.3)	2047.5 (762.2)	26.6 (2.9)	2449.7 (264.1)
Total cost		2303.1 (816.2)		2776.8 (399.4)

SD: standard deviation. GP: General Practitioner

Table S5: The odds of reporting at least one working day lost at 6 months after registration.

Patient characteristic	N (%)	Odds ratio (SE)	95 % CI	p-value
Age (years)				
18 - 29	15 (5.8%)	Reference		
30 - 49	90 (34.9%)	0.94 (0.67)	(0.23, 3.78)	0.930
50 - 64	135 (52.3%)	0.30 (0.21)	(0.08, 1.18)	0.084
65+	18 (7.0%)	0.26 (0.24)	(0.04, 1.56)	0.141
Gender				
Female	190 (73.6%)	Reference		
Male	68 (26.4%)	0.98 (0.34)	(0.50, 1.92)	0.953
IMD				
Quintile 2+	233 (90.3%)	Reference		
Quintile 1 (most deprived)	25 (9.7%)	0.76 (0.37)	(0.29, 1.97)	0.566
Months since baseline		0.94 (0.05)	(0.85, 1.03)	0.181
Having at least one working day lost at baseline				
No	118 (45.7%)	Reference		
Yes	140 (54.3%)	8.70 (2.68)	(4.75, 15.93)	<0.001

IMD: Index of multiple deprivation
