# Supplementary material for: Trajectories of functional limitations, health-related quality of life and societal costs in individuals with long COVID: a population-based longitudinal cohort study
Source: BMJ Open. 2024 Nov 13;14(11):e088538. doi: 10.1136/bmjopen-2024-088538 (PMC11574431; doi:10.1136/bmjopen-2024-088538)
Supplement: Supplementary file 2 [file bmjopen-2024-088538.reviewer_comments.pdf]

PEER REVIEW HISTORY

BMJ Open publishes all reviews undertaken for accepted manuscripts. Reviewers are asked to complete a checklist review form and are provided with free text boxes to elaborate on their assessment. These free text comments are reproduced below.

ARTICLE DETAILS

Title (Provisional)

Trajectories of functional limitations, health-related quality of life and societal costs in individuals with long COVID: a population-based longitudinal cohort study

Authors

Wang, Jiunn; Goodfellow, Henry; Walker, Sarah; Blandford, Ann; Pfeffer, Paul; Hurst, John R.; Sunkersing, David; Bradbury, Katherine; Robson, Chris; Henley, William; Gomes, Manuel

---

VERSION 1 - REVIEW

**Reviewer**	**1**
**Name**	**Kern, Winfried**
**Affiliation**	**Freiburg University**
**Date**	**09-Jun-2024**
**COI**	**none**

This work presents the results of a follow-up investigation via an app of self-reported outcomes among long Covid patients seen at several long Covid centers. The primary aim was to assess the trajectories of functional impairment over time (using the WSAS instrument). The design is appropriate, and the paper is well written.

Comments/questions:

- a limitation (not adressed) is that there are no data about the working situation of the participants (before COVID-19: not working/unemployed, part-time working, full-time working, employed or self-employed, or on training ect); it is unclear how this impacted on the results.

- it would be interesting to assess whether the changes in the subdomains of the WSAS were similar to each other

- it is not clear from the presentation whether time from acute index infection to registration might have had an impact on the improvement trajectories

- I missed an ethics statement

**Reviewer**	**2**
**Name**	**Takao, , Soshi**
**Affiliation**	**Okayama University Graduate School of Medicine**
**Date**	**25-Jun-2024**
**COI**	**n/a**

Major points

1. The target population was not clearly defined and described, especially the description of severity of the original COVID-19 is necessary. The bias due to digital literacy was discussed, however, the bias due to the different characteristics of care-seeking patients compared to non care-seeking patients should also be discussed. The point is that the subjects with poorer prognosis (i.e., care-seeking patients) were selected among the long COVID patients.

2. The relatively long observation period may allow stratified description of each virus variant, which may lead to different prognoses even for the same long COVID diagnosis.

3. The changes in each domain (e.g., work, home) of WSAS should be shown.

In relation to this point, 36% of subjects who were not working at all at 6 months should be analyzed separately (the mean of working day loss of 7.7 days is not suitable for reporting the summary measure, since it is expected that 1/3 of the subjects did not work and the rest did not contribute to working day loss).

Minor point

Line36 "DHI" should be spelled out.

VERSION 1 - AUTHOR RESPONSE

All the required statements were added at the end of the manuscript.

Reviewer: 1
Dr. Winfried Kern, Freiburg University
Comments to the Author:
This work presents the results of a follow-up investigation via an app of self-reported outcomes among long Covid patients seen at several long Covid centers. The primary aim was to assess the trajectories of functional impairment over time (using the WSAS instrument). The design is appropriate, and the paper is well written.

Comments/questions:
- a limitation (not addressed) is that there are no data about the working situation of the participants (before COVID-19: not working/unemployed, part-time working, full-time working, employed or self-employed, or on training etc); it is unclear how this impacted on the results.

Thanks, that's a pertinent point. The WSAS questionnaire includes a preliminary checkbox question to identify those individuals who are either retired or have chosen not to work for reasons unrelated to long COVID. The paper reported the results for those individuals who have not ticked this box. We revised the 'Population' sub-section (bottom of page 4) to reflect this point.

We were not able to distinguish between individuals in different types of job arrangements (part-time, full-time, self-employed), but our PPI work suggested that our sample includes a mix of employment types and hence, it is likely to be representative of the working-age population. We added a note to the limitations section (Page 14, paragraph 3).

- it would be interesting to assess whether the changes in the subdomains of the WSAS were similar to each other

Thanks for the suggestion. We now report the trajectories for the five WSAS components: ability to work, home management, social leisure activities, private leisure activities and close relationships (Online supplemental material, Figure S2). The trajectories for the individual components are very similar to that of the main score, i.e. slight decrease over time. It's worth noting that functional impairment in the individual's ability to work and enjoy social leisure activities remains moderately severe or worse over the 6-month follow up. We added these additional results to the 'Functional limitations' sub-section (Page 8).

- it is not clear from the presentation whether time from acute index infection to registration might have had an impact on the improvement trajectories

Thanks, this is a good point. The date of index COVID infection was not available in our study, and hence we were unable to evaluate whether time since acute index COVID infection has had an impact on long COVID recovery. We added this limitation to the 'Strengths and Limitations' sub-section in the Discussion (Page 15).

- I missed an ethics statement

An ethics statement is now included at the end of the manuscript.

Reviewer: 2
Dr. Soshi Takao, , Okayama University Graduate School of Medicine
Comments to the Author:
Major points
1. The target population was not clearly defined and described, especially the description of severity of the original COVID-19 is necessary. The bias due to digital literacy was discussed, however, the bias due to the different characteristics of care-seeking patients compared to non care-seeking patients should also be discussed. The point is that the subjects with poorer prognosis (i.e., care-seeking patients) were selected among the long COVID patients.

Many thanks for raising these helpful points. Given that the data were collected via the LWCR app, some clinical information, such as the severity of the acute COVID infection,

were not available in our study. Hence, we were unable to evaluate whether the severity of the COVID infection has had an impact on long COVID recovery. In addition, our focus was on the care-seeking population. Consequently, our findings may not be generalisable to non-care seeking patients, who are likely to be less impacted by the condition. We highlighted both limitations in the Discussion (Page 15, penultimate paragraph).

2. The relatively long observation period may allow stratified description of each virus variant, which may lead to different prognoses even for the same long COVID diagnosis.

Thanks for the suggestion. Information on SARS-COV-2 variants was not collected, so we were unable to assess its impact on long COVID trajectories. We refer to this point in 'Strengths and Limitations' sub-section in the Discussion (Page 15).

3. The changes in each domain (e.g., work, home) of WSAS should be shown.
In relation to this point, 36% of subjects who were not working at all at 6 months should be analyzed separately (the mean of working day loss of 7.7 days is not suitable for reporting the summary measure, since it is expected that 1/3 of the subjects did not work and the rest did not contribute to working day loss).

Thanks for these suggestions. We now report the trajectories for the five WSAS components: ability to work, home management, social leisure activities, private leisure activities and close relationships (online supplemental material, Figure S2). The trajectories for the individual components are very similar to that of the main score, i.e. slight decrease over time. It's worth noting that functional impairment in the individual's ability to work and enjoy social leisure activities remains moderately severe or worse over the 6-month follow up. We added these additional results to the 'Functional limitations' sub-section (Page 8).

We also reported a separate analysis for individuals (51 out of 141, 36%) who were not working at all at 6 months. The results, reported in online supplemental material table S4, suggested that these patients were somewhat worse off at 6 months compared to baseline. We added these additional results to the last section of the results section.

Minor point
Line36 "DHI" should be spelled out.

Thanks for spotting this, we revised this.

VERSION 2 - REVIEW

**Reviewer**	**2**
**Name**	**Takao, , Soshi**
**Affiliation**	**Okayama University Graduate School of Medicine**
**Date**	**18-Sep-2024**

COI **[None indicated].**

Even if the information on the SARS-COV-2 variant was not collected, the authors can perform a stratified analysis by the time period during which the variant strain was predominant.

VERSION 2 - AUTHOR RESPONSE

Reviewer: 2

Dr. Soshi Takao, Okayama University Graduate School of Medicine

Comments to the Author:

Even if the information on the SARS-COV-2 variant was not collected, the authors can perform a stratified analysis by the time period during which the variant strain was predominant.

We agree with the reviewer that a stratifying the analysis by the predominant SARS-COV-2 variant would be of interest. However, the predominant variant at a particular point in time does not necessarily matches the one that gave rise to long covid and that complicates any meaningful analysis. In addition, recent studies suggest that the prevalence and severity of long COVID were similar across SARS-COV-2 variants:

https://www.ncbi.nlm.nih.gov/pmc/articles/PMC9736973/

https://www.ncbi.nlm.nih.gov/pmc/articles/PMC10995878/

This limitation is acknowledged in the last paragraph of the Discussion section.
